# The Quiescent Period in Acute Abdominal Affections

**Published:** 1902-05-10

**Authors:** 


### THE QUIESCENT PERIOD IN ACUTE ABDOMINAL AFFECTIONS.

At the last meeting of the Medical Society a paper was read by Mr. A. H. Tubby on the Quiescent Period in the Course of Acute Abdominal Affections, in which he pointed out that after an acute onset in such cases there sometimes occurred a period of quiescence in which most of the symptoms might subside, the patient expressing himself as comfortable and free from pain; yet this might be but a prelude to an aggravation of the disease, with acute peritonitis and a fatal termination unless its significance were realised. This quiescent period was well marked in cases of injury to the intestines with or without rupture, intestinal perforation by ulceration, and other acute lesions, and particularly in acute appendicitis. The remission of symptoms was, however, never quite complete, and the importance of persistence of the altered proportion between pulse and temperature, of rigidity, of distension, and of vomiting was insisted upon. The main interest, however, both of Mr. Tubby's remarks and of the discussion which followed, lay in the support given to the important prognostic and indeed diagnostic significance of leucocytosis in abdominal affections. Leucocytosis was present in all these cases of latent mischief, and Mr. Tubby held that blood counts should not be omitted in any suspicious case. Leucocytosis was, he considered, of the utmost importance as an indication of the onset of acute peritonitis, and its presence in any marked degree afforded justifiable grounds for urging surgical interference without delay. Dr. J. H. Bryant also insisted upon the necessity of a blood examination in obscure abdominal cases where the question of operation arose, and he mentioned two cases, in one of which with a great excess of leucocytes a large abscess was found, while in the other in which there was but slight leucocytosis the inflammation subsided without suppuration. Mr. Jonathan Hutchinson, jun., on the other hand, thought that the evidence in regard to the value of leucocytosis as indicating operation was conflicting.

